# PP89 Health Technology Assessment: Access To Innovative Medicines In The USA And Organization For Economic Cooperation And Development

**DOI:** 10.1017/S0266462325103061

**Published:** 2025-12-29

**Authors:** Natalie LaHood

## Abstract

**Introduction:**

Health technology assessment (HTA) has increasingly shaped public health insurance reimbursement decisions across many countries during the last 10 years. As countries implement and expand their use of HTA in determining recommendations for reimbursement, patient access to new medicines is affected. This study analyzed the impact of HTA, and HTA expansion, on patient access to new medicines across OECD countries.

**Methods:**

New medicines were identified as new active substances approved by the European Medicines Agency, the United States Food and Drug Administration, or Japan’s Pharmaceuticals and Medical Devices Agency and launched globally between 1 January 2014, and 31 December 2023. Public health insurance reimbursement was determined by reviewing HTA recommendations by the appropriate HTA bodies and public reimbursement listings in each country. Patient utilization of new medicines was measured by estimating the number of patients treated with new medicines in each country, on a per capita basis, using sales and volume data adjusted for expected treatment durations.

**Results:**

During the past 10 years, HTA continued to play no official role in public health insurance reimbursement decisions for new medicines in the USA but played an expanding role in other OECD countries. New medicines increasingly launched first in the USA, with patients having access through public health insurance to 85 percent of new medicines in 2023. Patients in other OECD countries were far less likely to access new medicines within a year of first launch, and on average had access through public health insurance to less than a third of new medicines in 2023.

**Conclusions:**

The use of HTA contributes to patient access inequality across countries by limiting and delaying public health insurance reimbursement of new medicines. In the USA, which does not use HTA, patients have access to far more new medicines. Patients in countries where HTA bodies have become gatekeepers of determining which new medicines are cost effective tend to have worse access.

